# Molecular study of vitamin D metabolism-related single nucleotide polymorphisms in cardiovascular risk: a case-control study

**DOI:** 10.1007/s13105-025-01080-z

**Published:** 2025-04-16

**Authors:** José María Gálvez-Navas, Noelia Márquez-Pete, Madalena Paiva-Chaves, Susana Rojo-Tolosa, Laura Elena Pineda-Lancheros, Yasmin Cura, Cristina Membrive-Jiménez, Luciana Maria Marangoni-Iglecias, Andrea Fernández-Alonso, MCarmen Ramírez-Tortosa, Cristina Pérez-Ramírez, Alberto Jiménez-Morales

**Affiliations:** 1 Centro de Investigación Biomédica en Red en Epidemiología y Salud Pública (CIBERESP), Madrid, 28029 Spain; 2 Cancer Registry of Granada, Andalusian School of Public Health, Cuesta del Observatorio 4, Campus Universitario de Cartuja, Granada, 18011 Spain; 3 Instituto de Investigación Biosanitaria (ibs.GRANADA), Avda. de Madrid 15, Granada, 18012 Spain; 4 Department of Biochemistry and Molecular Biology II, Institute of Nutrition and Food Technology “José Mataix Verdú” (INYTA), Biomedical Research Centre (CIBM), Avda. del Conocimiento 19, Armilla, Granada, 18016 Spain; 5 Pharmacogenetics Unit, Pharmacy Department, University Hospital Virgen de las Nieves, Avda. de las Fuerzas Armadas 2, Granada, 18014 Spain; 6 Cardiology Department, University Hospital of Mérida, Avda. de Don Antonio Campo Hoyos 26, Mérida, Badajoz, 06800 Spain; 7 Pneumology Department, University Hospital Virgen de las Nieves, Avda. de las Fuerzas Armadas 2, Granada, 18014 Spain; 8 Departamento de Farmacia, Facultad de Ciencias, Universidad Nacional de Colombia, Carrera 30 nº 45-03, Edificio 476 Ciudad Universitaria de Bogotá, Bogotá, Colombia; 9 Federal University of Mato Grosso do Sul University Hospital, Avda. Sen. Filinto Müller, 355-Vila Ipiranga, Campo Grande-MS, Campo Grande, 79080-190 Brazil

**Keywords:** Cardiovascular diseases, Vitamin D, Single nucleotide polymorphisms, Risk, Biomarkers, Metabolism

## Abstract

Cardiovascular diseases (CVDs) constitute a major global health problem, being the leading cause of death. Several risk factors for CVDs have been identified, including tobacco use, unhealthy diet, and physical inactivity. However, the role of genetic factors in CVDs remains unclear. Recent studies suggest that vitamin D deficiency is associated with an increased risk of CVDs. Therefore, the aim of this study is to assess the impact of 13 single nucleotide polymorphisms (SNPs) located in genes involved in vitamin D metabolism (*VDR*, *GC*, *CYP27B1*, *CYP2R1*, and *CYP24A1*) on the risk of developing CVDs. A retrospective case-control study was conducted in 766 Caucasian individuals from southern Spain: 383 diagnosed with CVDs and 383 without cardiovascular complications, matched based on age and sex. The 13 SNPs were identified by real-time PCR using TaqMan™ probes at the Virgen de las Nieves University Hospital and the University of Granada. According to statistical analysis the allele G and genotype GG of the SNP *CYP2R1* rs10741657 and the allele C and CC genotype of the SNP *CYP27B1* rs3782130 are associated with a decreased risk of CVDs and diabetes in three of the five heritage models studied. Thus, it can be concluded that *CYP2R1* rs10741657 and *CYP27B1* rs3782130 could be used as risk biomarkers for CVDs in the future, although studies with a larger number of participants are needed.

**Supplementary Information:**

The online version contains supplementary material available at 10.1007/s13105-025-01080-z.

## Clinical trial number

Not applicable.

## Background

Cardiovascular diseases (CVDs) are disorders that affect the heart and blood vessels. Classic examples include coronary heart disease, which can lead to myocardial infarction, and cerebrovascular disease, which can result in stroke [1]. CVDs are the leading cause of mortality worldwide, representing a significant public health challenge. The World Health Organization estimates that 17.9 million lives are lost to CVDs each year. Moreover, one-third of these losses are premature deaths, occurring before the age of 70 [2, 3]. CVDs are the result of a complex interaction between genetic predisposition and non-genetic factors [4]. While extensive research and information on non-genetic risk factors, such as smoking or physical inactivity exist [5], the information on genetic predispositions is relatively limited.

Single Nucleotide Polymorphisms (SNPs) in genes involved in vitamin D metabolism have gained research interest due to their potential role in CVD risk, as vitamin D deficiency has emerged as a CV risk factor in recent decades. Scragg et al. first described a seasonal pattern of CVDs linked to low calcidiol levels [6], and since then, vitamin D receptors (VDR) have been identified in various CV cells. Thus, suggesting their involvement in key pathogenic pathways such as the renin-angiotensin-aldosterone system, aging, and cellular senescence, where it plays a regulatory role. Vitamin D also helps mitigate inflammation and oxidative stress associated with CVDs [7, 8]. Observational studies have reported an inverse correlation between vitamin D levels and CV events [9–11], linking deficiency to hypertension, cardiac hypertrophy, arterial stiffness, and myocardial infarction [9, 12, 13]. However, recent studies have not demonstrated a clear benefit of vitamin D supplementation in preventing CVDs [11, 14, 15], emphasizing the need for further research to identify patients who may benefit. Interestingly, emerging evidence suggests that VDR and GC polymorphisms could be risk factors for CVDs [16, 17], raising the possibility that individuals with specific genetic variants might respond favorably to vitamin D supplementation.

Vitamin D activity is regulated by a complex metabolic pathway involving five key genes. The *GC* gene (member of the specific-group component family) encodes for the vitamin D binding protein (VDBP), which transports vitamin D metabolites in the bloodstream, facilitating their distribution to target tissues [7]. The activation of vitamin D begins with hydroxylation in the liver, primarily catalyzed by the enzyme 1-α-hydroxylase (encoded in gene *CYP2R1*, member of the P450 cytochrome), which converts vitamin D into calcidiol (25(OH)D), the major circulating form [8]. In the kidney, 25-hydroxylase (coded by *CYP27B1*, member of the P450 cytochrome) further hydroxylates calcidiol to produce calcitriol (1,25(OH)₂D), the biologically active form that exerts regulatory effects on various physiological processes, including cardiovascular ones [18]. Once activated, calcitriol binds to the vitamin D receptor or VDR (coded by the *VDR* gene which belongs to the family of nuclear receptors), initiating a cascade of molecular interactions. Within target cells, VDR forms a heterodimer with the retinoid X receptor (RXR), allowing the complex to translocate to the nucleus and bind to vitamin D response elements (VDREs), thereby modulating the transcription of multiple genes. Among these, *OXTR*, *ETRB*, *ELN*, and *REN* play significant roles in cardiovascular pathogenesis by regulating vascular tone, arterial elasticity, and blood pressure [19]. To prevent excessive vitamin D activity, vitamin D3 24-hydroxylase (coded by *CYP24A1* gene, member of the P450 cytochrome) catalyzes hydroxylation reactions that convert calcitriol into calcitroic acid (1,24,25(OH)₃D), a water-soluble metabolite that is excreted in urine [20]. Notably, these five genes are highly polymorphic, and variations in their sequences may impact enzyme efficiency, receptor affinity and vitamin D transport, ultimately influencing individual susceptibility to CVDs [18].

On the basis of the foregoing, this study aims to assess the role of the 13 most studied SNPs located in genes involved in vitamin D metabolism as risk biomarkers for CVDs in a Caucasian population from the South of Spain.

## Methods

### Study subjects and clinical evaluation

The study was designed as a retrospective case-control analysis, including 766 participants of Caucasian origin, 383 cases diagnosed with CVDs and 383 controls matched by age and sex. The cases were diagnosed with CVDs at the University Hospital Virgen de las Nieves in Granada (Spain) between April 1998 and July 2022. The controls were healthy individuals aged 18 or older, recruited from the same hospital, who had resided in the same geographic area and had no personal history of CVDs nor vitamin D-related diseases. Participants’ recruitment started in April 2021 and ended in July 2022.

Accepting an alpha risk of 0.05 (5%) and a beta risk of 0.2 in a bilateral contrast, for a statistical power of 80% at least 88 subjects per group are required to detect a significant difference in the risk of cardiovascular disease according to the presence of the A/G alleles of the *CYP2R1* rs10741657 SNP. A loss-to-follow-up rate of 10% was estimated. The choice of the variable and the data used to calculate the sample size were based on previous studies of cardiovascular disease risk and the presence of SNPs in *CYP2R1* [21].

The study was conducted with the approval of the Research Ethics Committee CEIm/CEI of the province of Granada, in accordance with the Declaration of Helsinki (code: 0957-N-21). The subjects included in this study signed an informed consent for the collection and genetic analysis of saliva samples and their donation to the Andalusian Public Health System Biobank and the publication of the subsequent study results. Samples were identified with an alphanumeric code and treated confidentially.

Data collection was carried out through access to participants’ clinical history from the Public Health System of Andalusia (Sistema Andaluz de Salud, SAS) program Estación Clínica-Diraya. The study included clinical and sociodemographic variables such as sex, age, tobacco use, alcohol consumption, body mass index (BMI), dyslipidemia, high blood pressure (HBP), diabetes, and the type of CVD of the cases. The classification of study participants followed criteria outlined in previous articles [16, 22]. The cases were classified into different types of CVDs, including cardiac arrhythmias, cardiomyopathy, cerebrovascular disease, heart valve disease, congenital cardiopathy, coronary disease, and peripheral vascular disease.

### Genetic analyses

The DNA samples were extracted from saliva collected in 50 ml BD Falcon™ conical tubes (BD, Plymouth, UK) at the Virgen de las Nieves University Hospital, which is part of the Biobank of the SAS. The QIAamp^®^ DNA Mini Kit (Qiagen GmbH, Hilden, Germany) was used for DNA extraction and purification following the manufacturer’s specifications. The extracted DNA samples were stored at -80ºC in the Biobank of the University Hospital Virgen de las Nieves. The quality and quantity of DNA were measured using the NanoDrop 2000™ UV spectrophotometer (Thermo Fisher Scientific, Waltham, MA, USA) by determining the absorbance ratio at 260/280 nm (nanometers) and 260/230 (nm).

The selection of SNPs for the study was based on their frequency in the Iberian population and on previous publications in the scientific literature. Thirteen polymorphisms involved in vitamin D metabolism were identified using real-time polymerase chain reaction (PCR) with TaqMan™ probes (ABI Applied Biosystems, 7300 Real-Time PCR System). Table 1 presents the identified polymorphisms and their corresponding assay IDs. The genetic variants were analyzed with the QuantStudio™ 12 K Flex software (96 wells, Thermo Fisher Scientific, Waltham, MA, USA) at the Pharmacogenetics Unit at the Virgen de las Nieves University Hospital and the Department of Biochemistry and Molecular Biology II at the Institute of Nutrition and Food Technology (Biomedical Research Center, University of Granada). Final sample call rate was 99.99%. Followed quality control criteria were previously published [23].

**Table 1:** Single nucleotide polymorphisms in vitamin D metabolism genes and their TaqMan™ assay IDs

Gene	dbSNP ID	Location	Alleles	Assay ID
*VDR* (12q13.11)	rs1544410 (BsmI)	Intron 8	C > T	C___8716062_20
	rs11568820 (Cdx2)	Intron 1	C > T	C___2880808_10
	rs2228570 (FokI)	Exon 2	A > G	C__12060045_20
	rs7975232 (ApaI)	Intron 8	C > A	C__28977635_10
	rs731236 (TaqI)	Exon 9	A > G	C___2404008_10
*CYP27B1* (12q14.1)	rs4646536	Intron 6	A > G	C__25623453_10
	rs3782130	5’ Promoter	G > C	ANGZRHH*
	rs10877012	5’ UTR	G > T	C__26237740_10
	rs703842	3’ UTR	A > G	AN9HX2K*
*CYP24A1* (20q13.2)	rs6068816	Exon 6	C > T	C__25620091_20
	rs4809957	3’ UTR	A > G	C___3120981_20
*CYP2R1* (11p15.2)	rs10741657	5’ UTR	A > G	C___2958430_10
*GC* (4q13.3)	rs7041	Exon 11	A > C	C___3133594_30

*The single nucleotide polymorphisms were analyzed using custom assays by Thermo Fisher Scientific (Waltham, MA, USA). UTR: untranslated region

### Statistical analysis

The cases and controls were matched by age and sex in a 1:1 ratio using the score matching method through RStudio [24] in order to avoid potential sources of bias. Descriptive analysis was conducted using R software version 4.3.0 (R Foundation for Statistical Computing, Vienna, Austria) [25]. Quantitative variables were presented as mean ± standard deviation for normally distributed variables and median (25th and 75th percentiles) for variables with a non-normal distribution. The normality of variables was assessed using the Kolmogorov-Smirnov test with the Lillefors correction.

The Hardy-Weinberg equilibrium (HWE), haplotype frequency, as well as Lewontin’s D prime (D’) and linkage disequilibrium coefficient (r^2^), were calculated. Bivariate analysis was conducted to examine the relationship between the risk of developing CVDs and genetic polymorphisms. The analysis included genotypic, allelic, dominant, recessive, and additive models, and used the Pearson chi-square test, Fisher’s exact test, and logistic regression to provide Odds Ratios (OR) and the corresponding 95% confidence intervals (95% CI). The above-mentioned models are defined as genotypic (DD vs. dd and Dd vs. dd), allelic (d vs. D), dominant ((DD, Dd) vs. dd), recessive (DD vs. (Dd, dd)), and additive, where D refers to the major allele and d to the minor allele. The Bonferroni correction was used to prevent false associations in the analysis of multiple comparisons. Logistic regression models were used to determine the influence of potential confounding variables on the risk of CVD. Variables included in the multivariate analysis were those with *p*-value < 0.05 in the bivariate analysis. All analyses were considered significant at a *p*-value ≤ 0.05 and were conducted using the PLINK software as a tool for whole-genome association analysis and R version 4.3.0 (R Foundation for Statistical Computing, Vienna, Austria) [25, 26]. Linkage disequilibrium (LD) was assessed using Haploview 4.2 [27]. Haplotype analysis was performed using SNPStats, a web tool for association study analysis [28].

The analysis included an assessment of developing general CVD in the study population. Subsequently, a stratified analysis was performed according to the most frequent CVD within the sample, including arrhythmias and peripheral cardiovascular disease, in order to validate the results obtained. For this analysis, a logistic regression approach adjusted for potential confounders such as age, sex, and comorbidities was carried out.

## Results

### Characteristics of the study subjects

This study included a total of 766 individuals, consisting of 383 diagnosed cases of CVDs and 383 controls. Table 2 presents the demographic, clinical, and pathological characteristics of the study population.

**Table 2:** Clinicopathological characteristics of cardiovascular diseases in cases and controls

	Cases		Controls		χ²	*p*-value	OR	95%CI
	*N*	*n* (%)	*N*	*n* (%)				
Sex	383		383		0.532	0.466		
Men		161 (42.04)		171 (44.65)			1	-
Women		222 (57.96)		212 (55.35)			1.11	0.83–1.48
Age	383	73 (66–77)	383	72 (66–76)		0.211	1.01	0.99–1.03
Tobacco use	383		383		1.206	0.547		
Smokers		51 (13.32)		59 (15.40)			0.86	0.56–1.32
Former smokers		102 (26.63)		91 (23.76)			1.12	0.80–1.58
Non-smokers		214 (55.87)		215 (56.14)			1	-
Unknown		16 (4.18)		18 (4.70)			-	-
Alcohol consumption	383		383		3.599	0.165		
Drinker		67 (17.49)		47 (12.27)			1.31	0.87–1.99
Former drinker		8 (2.08)		13 (3.39)			0.56	0.22–1.37
Non-drinker		278 (74.53)		257 (67.10)			1	-
Unknown		30 (7.83)		66 (17.23)			-	-
Body Mass Index (kg/m^2^)	383	29.05 (26.00-33.30)	383	28.40 (25.00-31.40)				
Obese		126 (32.90)		79 (20.63)	2.313	0.315	1.40	0.88–2.24
Overweight		105 (27.41)		82 (21.41)			1.13	0.70–1.81
Normal (healthy weight)		59 (15.40)		52 (13.58)			1	-
Unknown		93 (24.28)		170 (44.89)			-	-
Dyslipidemia	383		383		0.86	0.363		
Yes		168 (43.98)		156 (40.73)			1.14	0.85–1.52
No		214 (56.02)		227 (59.27)			1	-
Unknown		1 (0.26)		0 (0.00)			-	-
High Blood Pressure	383		383		6.990	0.008		
Yes		230 (60.05)		187 (48.83)			1.47	1.11–1.96
No		149 (38.90)		196 (51.17)			1	-
Unknown		4 (1.04)		0 (0.00)			-	-
Diabetes	383		383		19.959	< 0.001		
Yes		132 (34.83)		78 (20.37)			2.09	1.51–2.90
No		247 (65.17)		305 (79.63)			1	-
Unknown		4 (1.04)		0 (0.00)			-	-
CVDs	383							
Cardiac arrhythmias		143 (37.34)						
Cardiomyopathy		21 (5.48)						
Cerebrovascular disease		20 (5.22)						
Heart valve disease		65 (16.97)						
Congenital cardiopathy		2 (0.52)						
Coronary disease		47 (12.27)						
Peripheral vascular disease		85 (22.19)						

Qualitative variables: number (percentage). Quantitative variables: Normal distribution: mean ± standard deviation. Non-normal distribution: P_50_ [P_25_, P_75_]. Shade means the *p*-value is significant. CVDs: cardiovascular diseases; OR: odds ratio; CI: confidence interval

Significant differences between cases and controls were found for High Blood Pressure (HBP) (OR = 1.47; 95% CI = 1.11–1.96; *p* = 0.008, No HBP vs. Yes HBP) and diabetes (OR = 2.09; 95% CI = 1.51–2.90; *p* < 0.001, No diabetes vs. Yes diabetes). However, there were no significant differences in age (*p* = 0.211) or sex (*p* = 0.466) (Table 2). In the stratified analysis, there were no significant differences between cardiac arrhythmias and peripheral vascular disease cases and controls for possible confounding variables.

### Allele and genotype frequencies of SNPs

**Fig. 1:**
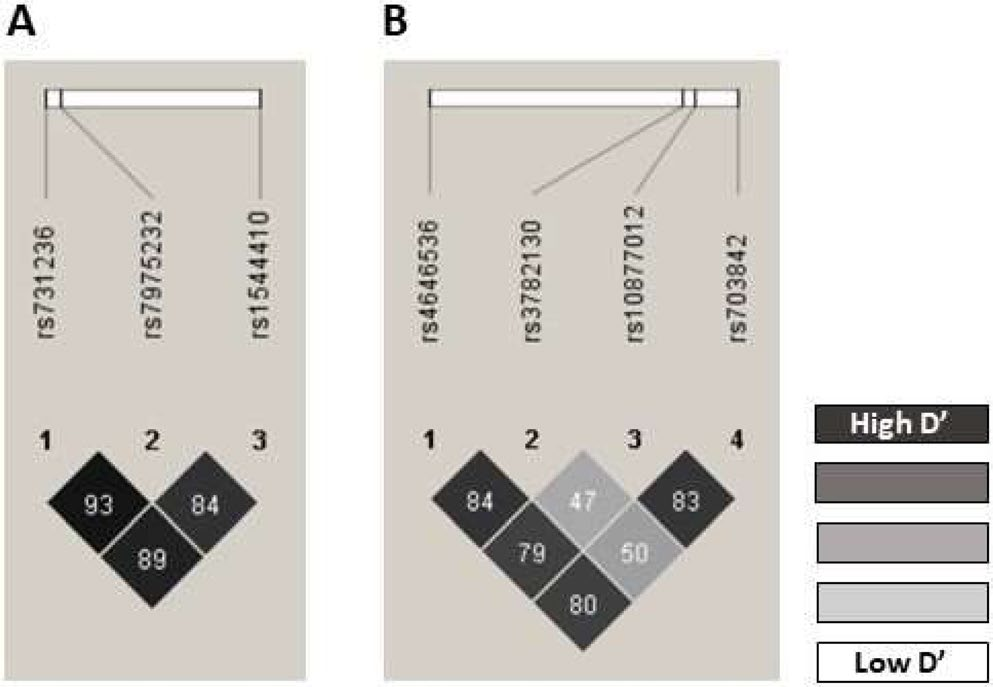
Linkage disequilibrium of the SNPs located in *VDR* and *CYP27B1* genes. Pairwise LD D’ plots for SNPs located in *VDR* and *CYP27B1* genes in the whole population. Numbers within the square are the D’ values expressed in percent: (**A**) pairwise LD D’ plots for 3 SNPs located in *VDR* gene, and (**B**) pairwise LD D’ plots for 4 SNPs located in *CYP27B1* gene. LD: linkage disequilibrium; SNPs: single nucleotide polymorphisms

The observed genotype frequencies in the control group did not deviate from the expected frequencies according to HWE, except for the SNPs *GC* rs7041 (p = 0.024) and *CYP27B1* rs4646536 (p > 0.001) (Table S1). However, no significant differences were found between the frequency of these polymorphisms and that reported for the Spanish Iberian population: *GC* rs7041 allele C: 0.5650 vs. 0.4628, p = 0.885; and *CYP27B1* rs4646536 allele G: 0.2900 vs. 0.2859, p = 0.995 [29]. The r^2^ and D’ values are presented in Table S2. The SNP pairs *VDR* rs1544410-rs7975232 (r^2^ = 0.4329, D’ = 0.8464); *VDR* rs1544410-rs731236 (r^2^ = 0.6841, D’ = 0.8937); *VDR* rs7975232-rs731236 (r^2^ = 0.4910, D’ = 0.9355); *CYP27B1* rs4646536-rs703842 (r^2^ = 0.3672, D’ = 0.8000); *CYP27B1* rs4646536-rs3782130 (r^2^ = 0.4220, D’ = 0.8395); *CYP27B1* rs4646536-rs10877012 (r^2^ = 0.3641, D’ = 0.7970); and *CYP27B1* rs703842-rs10877012 (r^2^ = 0.6686, D’ = 0.8334) were in strong LD based on D’ values (Fig. 1). All polymorphisms had a MAF greater than 5%, so none were excluded from the analysis (Table S3). The estimated haplotype frequencies are detailed in Table S4.

### Influence of single nucleotide polymorphisms on the risk of developing cardiovascular diseases

Table S5 shows the results of the bivariate analysis between the 13 SNPs involved in vitamin D metabolism included in the study and the risk of CVD for genotypic, allelic, recessive and additive models. Furthermore, for the SNPs with a significant p-value, OR and 95% CI have been calculated (Table 3).

The initial multivariate logistic regression analysis included the selected SNPs (*CYP2R1* rs10741657, *CYP27B1* rs4646536, and *CYP27B1* rs3782130), adjusted for HBP and diabetes. In this analysis, statistical significance was evaluated under different genetic inheritance models (genotypic, allelic, additive, dominant, and recessive). However, the *CYP27B1* rs4646536 polymorphism and HBP did not maintain significance across any model.

As a result, the final logistic regression model included only *CYP2R1* rs10741657 and *CYP27B1* rs3782130, adjusted for diabetes. Their association with CVD risk was assessed under all inheritance models. According to the genotypic model, individuals carrying the GG genotype for *CYP2R1* rs10741657 had a lower risk of developing CVD compared to those with the AA genotype (OR = 0.50; 95% CI = 0.31–0.79; *p* = 0.003). Similarly, individuals with the CC genotype for *CYP27B1* rs3782130 had a significantly lower risk compared to those with the GG genotype (OR = 0.09; 95% CI = 0.03–0.21; *p* < 0.001) (Table 4).

The recessive model confirmed these associations. The G allele of *CYP2R1* rs10741657 was associated with a reduced CVD risk (OR = 0.53; 95% CI = 0.34–0.81; *p* = 0.004; GG + GA vs. AA). Likewise, individuals with the CC genotype for *CYP27B1* rs3782130 had a lower risk compared to those carrying at least one G allele (OR = 0.08; 95% CI = 0.02–0.21; *p* < 0.001; CC vs. GG + GC). The additive model yielded consistent findings for both SNPs (OR = 0.74; 95% CI = 0.60–0.92; *p* = 0.006; G vs. A for *CYP2R1* rs10741657 and OR = 0.64; 95% CI = 0.50–0.82; *p* < 0.001; G vs. C for *CYP27B1* rs3782130) (Table 4). No associations were observed for the other genetic variants analyzed (Table S5).

To assess the robustness of the findings, statistical power was calculated based on the *CYP2R1* rs10741657 polymorphism. In the multivariate analysis, the recessive model estimated an OR of 0.50 (Table 4). Given a sample size of 383 cases and 383 controls, the power of the analysis was 99.7%. Similarly, for the *CYP27B1* rs3782130 SNP, the estimated OR in the additive model was 0.64 (Table 4). With a total sample of 766 individuals (1:1 case-control ratio), the power of the analysis was 89.3%.

Haplotype analysis was conducted for SNPs located on chromosome 12. The haplotypes CCAGAG and TAGGAG, which included six polymorphisms (rs1544410 (*VDR*), rs7975232 (*VDR*), rs731236 (*VDR*), rs4646536 (*CYP27B1*), rs703842 (*CYP27B1*), and rs10877012 (*CYP27B1*)), were associated with an increased risk of developing CVD. Specifically, individuals carrying the CCAGAG haplotype had a significantly higher risk (OR = 15.16; 95% CI = 2.01-114.41; *p* = 0.009), as did those carrying TAGGAG (OR = 2.85; 95% CI = 1.09–7.47; *p* = 0.033). Rare haplotypes (those with very low population frequency) were also linked to a higher risk of CVD (OR = 1.67; 95% CI = 1.13–2.48; *p* = 0.011). Conversely, individuals carrying the CAGAAG haplotype had a reduced risk (OR = 0.34; 95% CI = 0.12–0.99; *p* = 0.048) (Table 5).

A stratified analysis was performed for the most frequent CVDs in the study population: cardiac arrhythmias and peripheral vascular disease. For cardiac arrhythmias (*n* = 143 cases), significant associations were observed for *CYP27B1* rs4646536 under the genotypic (*p* = 0.009), recessive (*p* = 0.002), and allelic (*p* = 0.047) models, as well as for *CYP27B1* rs3782130 under the genotypic (*p* = 0.014) and recessive (*p* = 0.004) models. These associations remained significant after Bonferroni correction and adjustment for age and sex (Tables S6 and S7). For peripheral vascular disease (*n* = 85 cases), *CYP2R1* rs10741657 showed associations across all inheritance models. However, statistical significance was lost after Bonferroni correction (Tables S8 and S9).

**Table 3:** Influence of *CYP2R1* rs10741657, *CYP27B1* rs4646536 and *CYP27B1* rs3782130 gene polymorphisms on the risk of developing cardiovascular diseases

Models	Genotype	Cases [*n* (%)]	Controls [*n* (%)]	*p*-value^a^	OR (CI95%)	Adjusted *p*-value^b^
***CYP2R1 rs10741657***						
Genotypic	GG	149 (38.90)	172 (44.90)	0.012	0.51 (0.33–0.80)	0.156
	GA	165 (43.10)	170 (44.40)		0.58 (0.37–0.89)	
	AA	69 (18.00)	41 (10.70)		1	
Recessive	GG + GA	314 (81.98)	342 (89.30)	0.004	0.54 (0.35–0.82)	0.047
	AA	69 (18.02)	41 (10.70)		1	
Dominant	GG	149 (38.90)	172 (44.91)	0.092	0.78 (0.59–1.04)	1
	GA + AA	234 (61.10)	211 (55.09)		1	
Allelic	G	463 (60.44)	514 (67.10)	0.006	-	0.087
	A	303 (39.56)	252 (32.90)		-	
Additive	-	-	-	0.008	0.76 (0.62–0.93)	0.107
***CYP27B1*** **rs4646536**						
Genotypic	AA	222 (57.96)	197 (51.44)	< 0.001	1	0.002
	AG	134 (34.99)	122 (31.85)		0.97 (0.71–1.33)	
	GG	27 (7.05)	64 (16.71)		0.37 (0.22–0.60)	
Recessive	AA + AG	356 (92.95)	319 (83.29)	0.036	1	< 0.001
	GG	27 (7.05)	64 (16.71)		0.38 (0.23–0.60)	
Dominant	AA	222 (57.96)	197 (51.44)	0.069	1	0.853
	AG + GG	161 (42.04)	186 (48.56)		0.77 (0.58–1.02)	
Allelic	A	578 (75.46)	516 (67.36)	< 0.001		0.001
	G	188 (24.54)	250 (32.64)			
Additive	-	-	-	0.001	0.71 (0.58–0.88)	0.016
***CYP27B1*** **rs3782130**						
Genotypic	GG	236 (61.62)	214 (55.87)	< 0.001 ^c^	1	< 0.001
	GC	143 (37.34)	121 (31.59)		1.07 (0.79–1.45)	
	CC	4 (1.04)	48 (12.54)		0.08 (0.02–0.19)	
Recessive	GG + GC	379 (98.96)	335 (87.47)	< 0.001 ^c^	1	< 0.001
	CC	4 (1.04)	48 (12.53)		0.07 (0.02–0.18)	
Dominant	GG	236 (61.62)	214 (55.87)	0.123	1	1
	GC + CC	147 (38.38)	169 (44.13)		0.79 (0.59–1.05)	
Allelic	G	615 (80.29)	549 (71.67)	0.098		1
	C	151 (19.71)	217 (28.33)			
Additive	-	-	-	< 0.001 ^c^	0.63 (0.50–0.80)	< 0.001

^a^*p*-value for χ² test. ^b^*p*-value for Bonferroni correction. ^c^*p*-value for Fisher exact test. Shade means the value is significant. OR: odds ratio; CI: confidence interval

**Table 4:** Influence of clinical characteristics and *CYP2R1* rs10741657 and *CYP27B1* rs3782130 gene polymorphisms on the risk of developing cardiovascular diseases

	Genotypic				Recessive		Dominant		Additive	
	DD vs. dd		Dd vs. dd		DD + Dd vs. dd		DD vs. Dd + dd		DD = 2, Dd = 1, dd = 0	
	*p*-value	OR (95%IC)	*p*-value	OR (95%IC)	*p*-value	OR (95%IC)	*p*-value	OR (95%IC)	*p*-value	OR (95%IC)
*Diabetes*	< 0.001	1.98 (1.42–2.78)	< 0.001	1.98 (1.42–2.78)	< 0.001	1.98 (1.42–2.78)	< 0.001	2.09 (1.51–2.91)	< 0.001	2.07 (1.49–2.89)
*CYP2R1* *rs10741657*	0.003	0.50 (0.31–0.79)	0.013	0.56 (0.35–0.88)	0.004	0.53 (0.34–0.81)	0.082	0.77 (0.57–1.03)	0.006	0.74 (0.60–0.92)
***CYP27B1*** **rs3782130**	< 0.001	0.09 (0.03–0.21)	0.700	1.06 (0.78–1.45)	< 0.001	0.08 (0.02–0.21)	0.114	0.79 (0.59–1.06)	< 0.001	0.64 (0.50–0.82)
R^2^ Cox Snell = 0.039										
R^2^ Nagelkerke = 0.053										
Goodness of Fit: *p*-value < 0.001; χ² = 28.098										

Shade means the value is significant. OR: odds ratio; CI: confidence interval

**Table 5:** Influence of haplotypes formed by 6 SNPs located on chromosome 12 on the risk of developing cardiovascular diseases

ID	rs1544410 (VDR)	rs7975232 (VDR)	rs731236 (VDR)	rs4646536 (CYP27B1)	rs703842 (CYP27B1)	rs10877012 (CYP27B1)	Freq.	OR (95% CI)	*p*-value
1	C	C	A	A	A	G	0.306	1.00	-
2	T	A	G	A	A	G	0.216	1.17 (0.85–1.60)	0.330
3	T	A	G	G	G	T	0.089	0.90 (0.58–1.37)	0.610
4	C	A	A	A	A	G	0.085	1.21 (0.80–1.84)	0.360
5	C	C	A	G	G	T	0.072	1.13 (0.67–1.91)	0.640
6	C	C	A	G	A	G	0.034	15.16 (2.01-114.41)	0.009
7	T	A	G	G	A	G	0.022	2.85 (1.09–7.47)	0.033
8	T	C	A	A	A	G	0.019	0.98 (0.49–1.98)	0.960
9	C	A	A	G	G	T	0.017	1.59 (0.55–4.64)	0.400
10	C	A	G	A	A	G	0.017	0.34 (0.12–0.99)	0.048
11	T	A	A	A	A	G	0.015	1.74 (0.70–4.25)	0.230
12	C	C	A	A	G	G	0.011	1.04 (0.39–2.74)	0.940
Rare	*	*	*	*	*	*	0.009	1.67 (1.13–2.48)	0.011

Freq.: haplotype frequency in the whole population. * Refers to those rare haplotypes with haplotype frequency < 0.01 in the whole population, as there is no symbol to identify this group. Shade means the *p*-value is significant. OR: odds ratio; CI: confidence interval; SNPs: single nucleotide polymorphisms

## Discussion

CVDs encompass a broad range of pathologies affecting multiple organs and tissues, representing a major global health concern [30]. While environmental factors such as tobacco use, physical inactivity, and unhealthy diet have been well established as contributors to CVD onset [31], the role of genetic factors remains less understood. Studies suggest that low vitamin D levels in the blood may promote CVD development [32–34]. However, few studies have assessed the role SNPs in genes involved in vitamin D metabolism, specifically *VDR*,* CYP27B1*,* CYP2R1*,* CYP24A1*, and *GC*. This study comprehensively analyzes SNPs in these genes, making it the first of its kind. Our findings indicate that *CYP27B1* rs3782130 and *CYP2R1* rs10741657 may be involved in CVD development. Given the heterogeneous nature of CVDs, we performed a stratified analysis for the most prevalent conditions in our study population, namely cardiac arrhythmias and peripheral vascular disease. Notably, the results of this stratified analysis align with our overall findings for general CVD risk.

The *CYP2R1* gene, located on chromosome 11 (11p15.2), encodes the enzyme CYP2R1 (25-hydroxylase), which hydroxylates vitamin D precursors to form calcidiol [32, 35]. Previous studies have associated *CYP2R1* rs10741657 with variations in serum vitamin D levels, but findings have been contradictory [36, 37]. Moreover, its effect on CVD risk has been sparsely studied [36–38]. Our study suggests that individuals carrying the G allele of *CYP2R1* rs10741657 have a lower risk of developing CVDs, likely due to its role in maintaining enzyme activity and preserving vitamin D metabolism. These results contrast with previous studies, which associated the G allele with both higher vitamin D levels and increased CVD risk [36, 37]. For instance, Hassanein et al. found that Caucasian (Egyptian) individuals carrying the G allele had an increased risk of coronary artery disease (CAD) (OR = 2.55; 95% CI = 1.44–4.52; *p* = 0.002; G vs. A) [36]. Furthermore, Türkanoğlu Özçelik et al. reported a higher risk of ischemic stroke in Caucasian (Turkish) hypertensive, diabetic, smoking, and obese individuals (OR = 3.40; 95% CI = 2.12–5.51; *p* < 0.01; GG + GA vs. AA), (OR = 2.80; 95% CI = 1.58–4.98; *p* < 0.01; GG + GA vs. AA), (OR = 2.23; 95% CI = 1.20–4.14; *p* = 0.01; GG + GA vs. AA), and (OR = 4.8; 95% CI = 2.11–10.98; *p* < 0.01; GG + GA vs. AA), respectively] [37]. In contrast, a study by Brøndum-Jacobsen et al. in a large Danish cohort found no significant association between *CYP2R1* rs10741657 and ischemic heart disease or myocardial infarction risk, although lower vitamin D levels were linked to a higher CVD risk (*p* = 0.96) [38]. The discrepancies between these findings and our results may be attributed to differences in sample size, vitamin D measurement methods and study design.

The *CYP27B1* gene, located on chromosome 12 (12q14.1), encodes the enzyme CYP27B1 (1-α-hydroxylase), responsible for converting calcidiol into its active form, calcitriol [32, 39]. We analyzed SNPs rs4646536, rs3782130, rs10877012, and rs703842. Our study found that the *CYP27B1* rs3782130-C allele is associated with a reduced risk of CVDs. Notably, this is the first study to evaluate this SNP in relation to CVD risk, as previous studies have focused on its potential role in neoplastic diseases [40]. Further research is needed to confirm this association. Among the other SNPs analyzed, only *CYP27B1* rs4646536 has been previously studied for its relation to CVDs. Our bivariate analysis suggested that the GG genotype of *CYP27B1* rs4646536 might be associated with a lower CVD risk, but this was not supported by multivariate logistic regression **(**Table 3**)**. These findings align with Shen et al. (Caucasian subject) study, which reported no association between *CYP27B1* rs4646536 and coronary artery calcification (CAC) in a large Caucasian cohort (*p* > 0.05) [41].

The *VDR* gene, located on chromosome 12 (12q13.11), encodes the vitamin D receptor, which binds to vitamin D response elements to mediate its biological activity [32, 42]. We analyzed five common *VDR* SNPs: BsmI (rs1544410), TaqI (rs721236), ApaI (rs7975232), FokI (rs2228570), and Cdx2 (rs11568820), but found no significant associations with CVD risk (Table S5). However, the FokI polymorphism showed a borderline association in the recessive (*p* = 0.051) and allelic (*p* = 0.087) models. These results differ from previous studies by our group, where the FokI-AA genotype was linked to increased CVD risk in a Spanish cohort (OR = 2.30; 95% CI = 1.06–5.37; *p* = 0.043; AA vs. **GG)** [16]. Similarly, Nakhl et al. associated the G allele of VDR FokI with CVD risk in a Mediterranean population [43]. Conversely, other studies, such as Huzmeli et al. [44] and a meta-analysis by Shen et al. (2010) and Tabei et al. (2021) [41, 45], found no significant association between FokI and CVDs. These discrepancies may stem from differences in sample size, ethnicity and study design.

We also evaluated *GC* rs7041, *CYP24A1* rs6068816, and *CYP24A1* rs4809957 but found no significant associations with CVD risk (Table S5). Our results align with those of Daffara et al., who found no link between *GC* rs7041 and CAD risk in an Italian cohort (*p* = 0.81) [46]. However, a US study reported an increased heart failure risk in individuals carrying the *GC* rs7041-CC genotype (OR = 1.67; 95% CI = 1.15–2.24; *p* < 0.01; CC vs. AC + AA) [47]. Additionally, Kiani et al. suggested that the *GC* rs7041-C allele may offer protection against valvopathies in caucasian population (Iran) (OR = 0.67; 95% CI = 0.51–0.87; *p* = 0.003; AC + CC vs. AA) [48]. The effect of *CYP24A1* rs6068816 on CVDs has only been studied in an Asian population, where it was associated with CAD risk (*p* = 0.014, T vs. C) [49]. Given ethnic differences in allele frequencies, additional studies in diverse populations are necessary. This is the first study to evaluate *CYP24A1* rs4809957 in CVD risk, highlighting the need for further research.

This study has several limitations. First, its retrospective design limits data availability, as some relevant information was absent from participants’ medical histories. Second, the sample size and heterogeneity, particularly in case distribution, may reduce statistical power and mask certain associations. Moreover, the stratified analysis further reduces the sample size, potentially limiting the detection of significant associations. Additionally, serum vitamin D levels were not measured due to the retrospective nature of the study and the lack of such data in medical records. This absence prevents us from directly correlating SNPs with vitamin D status. However, despite these limitations, our results remain robust after Bonferroni correction, confirming associations between *CYP2R1* rs10741657 and *CYP27B1* rs3782130 with general CVD risk, as well as *CYP27B1* rs3782130 and *CYP27B1* rs4646536 with cardiac arrhythmias. A key strength of our study is the careful selection of cases from the Virgen de las Nieves University Hospital, ensuring diagnostic accuracy. Controls were matched for geographic origin, age and sex, therefore enhancing internal validity. Nonetheless, given the study’s limitations and the need for more scientific evidence, our findings should be interpreted with caution. Future studies focusing on specific CVD subtypes are necessary to confirm these associations.

## Conclusions

The study results suggest that the *CYP2R1* rs10741657-G and *CYP27B1* rs3782130-T alleles could be associated with a lower risk of developing CVDs in Caucasian individuals in Spain. In contrast, no association was found between the *VDR* BsmI, *VDR* Cdx2, *VDR* FokI, *VDR* ApaI, *VDR* TaqI, *GC* rs7041, *CYP27B1* rs4646536, *CYP27B1* rs10877012, *CYP27B1* rs703842, *CYP24A1* rs6068816, and *CYP24A1* rs4809957 polymorphisms and the risk of developing CVDs. Further studies are needed to finally include these SNPs in clinical practice.

## Electronic supplementary material

Below is the link to the electronic supplementary material.

Supplementary Material 1

## Data Availability

Data is provided within the manuscript or supplementary information files.

## Abbreviations

BMI: Body mass index
Calcidiol: 25(OH)D
CAC: Coronary arterial calcification
CAD: Coronary artery disease
Calcitriol: 1,25(OH)2D
CI: Confidence interval
CV: Cardiovascular
CVDs: Cardiovascular diseases
D’: Lewontin’s D prime
DNA: Desoxyribonucleic acid
HBP: High blood pressure
HWE: Hardy-Weinberg equilibrium
LD: Linkage disequilibrium
MAF: Minor allele frequency
OR: Odds ratio
r2: Linkage disequilibrium coefficient
RXR: Retinoid X receptor
SNP: Single nucleotide polymorphism
UTR: Untranslated region
VDBP: Vitamin D binding protein
VDR: Vitamin D receptor
VDRE: Vitamin D response element
