# Supplementary material for: Molecular study of vitamin D metabolism-related single nucleotide polymorphisms in cardiovascular risk: a case-control study
Source: J Physiol Biochem. 2025 Apr 16;81(2):347–57. doi: 10.1007/s13105-025-01080-z (PMC12279573; doi:10.1007/s13105-025-01080-z)
Supplement: Supplementary file 1 — Supplementary Material 1 [file 13105_2025_1080_MOESM1_ESM.zip › Table S1.docx]

**Table S1.** Hardy-Weinberg Equilibrium test results in the whole population, the case group, and the control group, respectively.

| **Chr** | **SNP** | **Gene** | **Sample** | **Minor Allele** | **Major Allele** | **Genotype counts** | **Observed heterozygosity** | **Expected heterozygosity** | ***p*-value** |
| --- | --- | --- | --- | --- | --- | --- | --- | --- | --- |
| 4 | rs7041 | *GC* | ALL | T | G | 153/403/210 | 0.5261 | 0.4972 | 0.1268 |
|  |  |  | AFF | T | G | 78/189/116 | 0.4935 | 0.4951 | 1 |
|  |  |  | UNAFF | T | G | 75/214/94 | 0.5587 | 0.4988 | 0.02404 |
| 11 | rs10741657 | *CYP2R1* | ALL | A | G | 110/335/321 | 0.4373 | 0.4621 | 0.1379 |
|  |  |  | AFF | A | G | 69/165/149 | 0.4308 | 0.4782 | 0.0546 |
|  |  |  | UNAFF | A | G | 41/170/172 | 0.4439 | 0.4415 | 1 |
| 12 | rs731236 | *VDR* | ALL | C | T | 121/385/287 | 0.4674 | 0.4765 | 0.5960 |
|  |  |  | AFF | C | T | 65/180/138 | 0.4700 | 0.4818 | 0.6713 |
|  |  |  | UNAFF | C | T | 56/178/149 | 0.4648 | 0.4705 | 0.8282 |
| 12 | rs7975232 | *VDR* | ALL | C | A | 186/365/215 | 0.4765 | 0.4993 | 0.2185 |
|  |  |  | AFF | C | A | 91/184/108 | 0.4804 | 0.4990 | 0.4742 |
|  |  |  | UNAFF | C | A | 95/181/107 | 0.4726 | 0.4995 | 0.3065 |
| 12 | rs1544410 | *VDR* | ALL | A | G | 136/356/274 | 0.4648 | 0.4838 | 0.2956 |
|  |  |  | AFF | A | G | 70/174/139 | 0.4543 | 0.4838 | 0.2454 |
|  |  |  | UNAFF | A | G | 66/306/415 | 0.4752 | 0.4838 | 0.7515 |
| 12 | rs2228570 | *VDR* | ALL | T | C | 111/318/337 | 0.4151 | 0.4565 | 0.01401 |
|  |  |  | AFF | T | C | 65/156/162 | 0.4073 | 0.4679 | 0.01199 |
|  |  |  | UNAFF | T | C | 46/162/175 | 0.4230 | 0.4433 | 0.3587 |
| 12 | rs11568820 | *VDR* | ALL | A | G | 45/306/415 | 0.3995 | 0.3833 | 0.2595 |
|  |  |  | AFF | A | G | 24/150/209 | 0.3916 | 0.3833 | 0.7897 |
|  |  |  | UNAFF | A | G | 21/156/206 | 0.4073 | 0.3833 | 0.2854 |
| 12 | rs4646536 | *CYP27B1* | ALL | G | A | 91/256/419 | 0.3342 | 0.4083 | 0.000934 |
|  |  |  | AFF | G | A | 27/134/222 | 0.3499 | 0.3704 | 0.2716 |
|  |  |  | UNAFF | G | A | 64/122/197 | 0.3185 | 0.4397 | 0.000117 |
| 12 | rs3782130 | *CYP27B1* | ALL | C | G | 52/264/450 | 0.3446 | 0.3650 | 0.000172 |
|  |  |  | AFF | C | G | 4/143/236 | 0.3734 | 0.3165 | 0.0262 |
|  |  |  | UNAFF | C | G | 48/121/214 | 0.3159 | 0.4061 | 0.09313 |
| 12 | rs10877012 | *CYP27B1* | ALL | T | G | 51/263/450 | 0.3433 | 0.3657 | 0.09313 |
|  |  |  | AFF | T | G | 25/139/219 | 0.3629 | 0.3717 | 0.6798 |
|  |  |  | UNAFF | T | G | 28/124/231 | 0.3228 | 0.3595 | 0.06347 |
| 12 | rs703842 | *CYP27B1* | ALL | C | T | 52/263/451 | 0.3433 | 0.3643 | 0.1128 |
|  |  |  | AFF | C | T | 25/140/218 | 0.3655 | 0.3730 | 0.6821 |
|  |  |  | UNAFF | C | T | 27/123/233 | 0.3211 | 0.3554 | 0.0619 |
| 20 | rs4809957 | *CYP24A1* | ALL | G | A | 40/257/469 | 0.3355 | 0.3432 | 0.5282 |
|  |  |  | AFF | G | A | 15/134/234 | 0.3499 | 0.3365 | 0.5433 |
|  |  |  | UNAFF | G | A | 25/123/235 | 0.3211 | 0.3497 | 0.1092 |
| 20 | rs6068816 | *CYP24A1* | ALL | T | C | 25/154/587 | 0.2010 | 0.2309 | 0.000825 |
|  |  |  | AFF | T | C | 13/67/303 | 0.1749 | 0.2133 | 0.001237 |
|  |  |  | UNAFF | T | C | 12/87/284 | 0.2272 | 0.2478 | 0.1010 |
| Chr: Chromosome; ALL: All population; AFF: Case group; UNAFF: Control group; SNP: Single Nucleotide Polymorphisms | | | | | | | | | |
