# Supplementary material for: Molecular study of vitamin D metabolism-related single nucleotide polymorphisms in cardiovascular risk: a case-control study
Source: J Physiol Biochem. 2025 Apr 16;81(2):347–57. doi: 10.1007/s13105-025-01080-z (PMC12279573; doi:10.1007/s13105-025-01080-z)
Supplement: Supplementary file 1 — Supplementary Material 1 [file 13105_2025_1080_MOESM1_ESM.zip › Table S2.docx]

Table S2. Linkage desequilibrium test results in the whole population.

| **Chr** | **Position**  **(base pair)** | **SNP1** | **Gene** | **Chr** | **Position (base pair)** | **SNP2** | **Gene** | **R^2^** | **D’** |
| --- | --- | --- | --- | --- | --- | --- | --- | --- | --- |
| 12 | 47846052 | rs1544410 | *VDR* | 12 | 47845054 | rs7975232 | *VDR* | 0.432937 | 0.846416 |
| 12 | 47846052 | rs1544410 | *VDR* | 12 | 47844974 | rs731236 | *VDR* | 0.684162 | 0.893718 |
| 12 | 47845054 | rs7975232 | *VDR* | 12 | 47844974 | rs731236 | *VDR* | 0.491038 | 0.935597 |
| 12 | 57764205 | rs4646536 | *CYP27B1* | 12 | 57768956 | rs703842 | *CYP27B1* | 0.367253 | 0.800004 |
| 12 | 57764205 | rs4646536 | *CYP27B1* | 12 | 57768115 | rs3782130 | *CYP27B1* | 0.422053 | 0.839520 |
| 12 | 57764205 | rs4646536 | *CYP27B1* | 12 | 57768302 | rs10877012 | *CYP27B1* | 0.364162 | 0.797044 |
| 12 | 57768956 | rs703842 | *CYP27B1* | 12 | 57768302 | rs10877012 | *CYP27B1* | 0.668646 | 0.833445 |
| Chr: Chromosome; SNP: Single Nucleotide Polymorphism. | | | | | | | | | |
