# Supplementary material for: Molecular study of vitamin D metabolism-related single nucleotide polymorphisms in cardiovascular risk: a case-control study
Source: J Physiol Biochem. 2025 Apr 16;81(2):347–57. doi: 10.1007/s13105-025-01080-z (PMC12279573; doi:10.1007/s13105-025-01080-z)
Supplement: Supplementary file 1 — Supplementary Material 1 [file 13105_2025_1080_MOESM1_ESM.zip › Table S3.docx]

Table S3. Minor allele frequency of 13 SNPs in the whole population.

| **Chr** | **SNP** | **Gene** | **Minor Allele** | **Major Allele** | **MAF** |
| --- | --- | --- | --- | --- | --- |
| 4 | rs7041 | *GC* | T | G | 0.4628 |
| 11 | rs10741657 | *CYP2R1* | A | G | 0.3623 |
| 12 | rs731236 | *VDR* | C | T | 0.3916 |
| 12 | rs7975232 | *VDR* | C | A | 0.4811 |
| 12 | rs1544410 | *VDR* | A | G | 0.4099 |
| 12 | rs2228570 | *VDR* | T | C | 0.3525 |
| 12 | rs11568820 | *VDR* | A | G | 0.2585 |
| 12 | rs4646536 | *CYP27B1* | G | A | 0.2859 |
| 12 | rs3782130 | *CYP27B1* | C | G | 0.2402 |
| 12 | rs10877012 | *CYP27B1* | T | G | 0.2409 |
| 12 | rs703842 | *CYP27B1* | C | T | 0.2396 |
| 20 | rs4809957 | *CYP24A1* | G | A | 0.2200 |
| 20 | rs6068816 | *CYP24A1* | T | C | 0.1332 |
| Chr: Chromosome; MAF: Minor Allele Frequency; SNP: Single Nucleotide Polymorphism. | | | | | |
