# Supplementary material for: Molecular study of vitamin D metabolism-related single nucleotide polymorphisms in cardiovascular risk: a case-control study
Source: J Physiol Biochem. 2025 Apr 16;81(2):347–57. doi: 10.1007/s13105-025-01080-z (PMC12279573; doi:10.1007/s13105-025-01080-z)
Supplement: Supplementary file 1 — Supplementary Material 1 [file 13105_2025_1080_MOESM1_ESM.zip › Table S4.docx]

|  | rs1544410 (*VDR*) | rs7975232 (*VDR*) | rs731236 (*VDR*) | rs4646536 (*CYP27B1*) | rs703842 (*CYP27B1*) | rs10877012 (*CYP27B1*) | ALL | AFF | UNAFF | Cumulative  frequency |
| --- | --- | --- | --- | --- | --- | --- | --- | --- | --- | --- |
| 1 | G | C | T | A | T | G | 0.3216 | 0.3437 | 0.317 | 0.3216 |
| 2 | A | A | C | A | T | G | 0.2349 | 0.2458 | 0.2287 | 0.5566 |
| 3 | A | A | C | G | C | T | 0.1134 | 0.1115 | 0.1132 | 0.6699 |
| 4 | G | A | T | A | T | G | 0.1011 | 0.0941 | 0.102 | 0.771 |
| 5 | G | C | T | G | C | T | 0.0738 | 0.071 | 0.0728 | 0.8448 |
| 6 | A | C | T | A | T | G | 0.0199 | 0.0165 | 0.0213 | 0.8647 |
| 7 | G | A | T | G | C | T | 0.0161 | 0.0181 | 0.0158 | 0.8809 |
| 8 | G | A | C | A | T | G | 0.0154 | 0.0174 | 0.0131 | 0.8963 |
| 9 | A | A | T | A | T | G | 0.0144 | 0.0184 | 0.0123 | 0.9107 |
| 10 | G | C | T | G | T | G | 0.0073 | NA | 0.0112 | 0.9179 |
| 11 | G | A | C | G | C | T | 0.0071 | 0.0173 | 0.0031 | 0.925 |
| 12 | G | C | T | G | C | G | 0.0059 | 0 | 0.0072 | 0.9309 |
| 13 | A | C | C | A | T | G | 0.0059 | 0.0046 | 0.0049 | 0.9368 |
| 14 | G | C | T | A | C | T | 0.0059 | 0 | 0.0072 | 0.9427 |
| 15 | A | A | C | G | T | G | 0.0057 | 0.0064 | 0.0052 | 0.9484 |
| 16 | G | C | T | A | C | G | 0.0053 | 0.0044 | 0.0056 | 0.9537 |
| 17 | A | A | C | A | C | G | 0.0045 | 0.0022 | 0.0054 | 0.9583 |
| 18 | A | C | T | G | C | T | 0.0045 | 0.0031 | 0.0054 | 0.9628 |
| 19 | G | C | T | G | T | T | 0.0043 | NA | 0.0066 | 0.967 |
| 20 | A | A | C | G | C | G | 0.0042 | NA | 0.0064 | 0.9712 |
| 21 | G | C | T | A | T | T | 0.004 | 0.0021 | 0.0048 | 0.9752 |
| 22 | A | A | T | G | C | T | 0.0039 | 0.0026 | 0.0043 | 0.9791 |
| 23 | G | A | T | A | T | T | 0.0035 | NA | 0.0059 | 0.9826 |
| 24 | A | A | C | G | T | T | 0.0034 | NA | 0.0049 | 0.9859 |
| 25 | G | C | C | A | T | G | 0.0022 | NA | 0.0032 | 0.9881 |
| 26 | G | A | T | G | C | G | 0.002 | 0.0041 | 0.0018 | 0.9901 |
| 27 | G | A | T | A | C | T | 0.0018 | 0.0021 | 0.0003 | 0.9919 |
| 28 | A | A | C | A | T | T | 0.0016 | NA | 0.0026 | 0.9935 |
| 29 | A | C | T | A | C | G | 0.001 | NA | 0 | 0.9945 |
| 30 | G | A | T | G | T | T | 9e-04 | NA | 0.0013 | 0.9954 |
| 31 | G | A | C | A | C | T | 8e-04 | 0.0022 | NA | 0.9962 |
| 32 | G | C | C | A | C | G | 8e-04 | NA | 0.0012 | 0.9969 |
| 33 | G | C | C | G | T | T | 7e-04 | NA | 0.0011 | 0.9976 |
| 34 | A | A | T | G | T | G | 7e-04 | 0.0021 | NA | 0.9983 |
| 35 | A | A | T | G | C | G | 6e-04 | 0.002 | NA | 0.9989 |
| 36 | G | C | C | G | C | T | 5e-04 | NA | 0.001 | 0.9994 |
| 37 | G | A | T | A | C | G | 5e-04 | NA | 0.002 | 0.9999 |
| 38 | A | A | T | A | C | G | 1e-04 | 0.0018 | NA | 1 |
| 39 | A | C | C | G | C | T | 0 | 0.0025 | NA | 1 |
| 40 | A | C | C | A | C | G | 0 | NA | 0.0014 | 1 |
| 41 | A | A | C | A | C | T | 0 | 0.0041 | NA | 1 |
| ALL: All population; AFF: Case group; UNAFF: Control group; SNP: Single Nucleotide Polymorphism; NA: Not Applicable. | | | | | | | | | | |

Table S4. Haplotype frequency estimation for 6 SNPs located on Chromosome 12 in the whole population.
