# Supplementary material for: Molecular study of vitamin D metabolism-related single nucleotide polymorphisms in cardiovascular risk: a case-control study
Source: J Physiol Biochem. 2025 Apr 16;81(2):347–57. doi: 10.1007/s13105-025-01080-z (PMC12279573; doi:10.1007/s13105-025-01080-z)
Supplement: Supplementary file 1 — Supplementary Material 1 [file 13105_2025_1080_MOESM1_ESM.zip › Table S5.docx]

**Table S5. Association of 13 SNPs with risk of cardiovascular disease.**

| **SNP** | **Gene** | **Minor**  **Allele** | **Major**  **Allele** | **Model** | **Cases** | **Controls** | **χ²** | ***p*-value**  **(χ² test)** | ***p*-value**  **(FET)** | **Adjusted *p*-value^a^** |
| --- | --- | --- | --- | --- | --- | --- | --- | --- | --- | --- |
| rs7041 | *GC* | T | G | Genotypic | 78/189/116 | 75/214/94 | 3.914 | 0.1412 | 0.1387 | 1 |
|  |  |  |  | Additive | 345/421 | 364/402 | 1.006 | 0.3158 | 0.3158 | 1 |
|  |  |  |  | Allelic | 345/421 | 364/402 | 0.9478 | 0.3303 | 0.3564 | 1 |
|  |  |  |  | Dominant | 267/116 | 289/94 | 3.175 | 0.07476 | 0.08882 | 0.8971 |
|  |  |  |  | Recessive | 78/305 | 75/308 | 0.07351 | 0.7863 | 0.8566 | 1 |
| rs10741657 | *CYP2R1* | A | G | Genotypic | 69/165/149 | 41/170/172 | 8.85 | 0.01197 | 0.01176 | 0.1556 |
|  |  |  |  | Additive | 303/463 | 252/514 | 6.975 | 0.008263 | 0.008263 | 0.1074 |
|  |  |  |  | Allelic | 303/463 | 252/514 | 7.349 | 0.006711 | 0.00784 | 0.0872 |
|  |  |  |  | Dominant | 234/149 | 211/172 | 2.837 | 0.09213 | 0.1071 | 1 |
|  |  |  |  | Recessive | 69/314 | 41/342 | 8.322 | 0.003916 | 0.00523 | 0.04699 |
| rs731236 | *VDR* | C | T | Genotypic | 65/180/138 | 56/178/149 | 1.102 | 0.5763 | 0.5838 | 1 |
|  |  |  |  | Additive | 310/456 | 290/476 | 1.075 | 0.2998 | 0.2998 | 1 |
|  |  |  |  | Allelic | 310/456 | 290/476 | 1.096 | 0.2952 | 0.32 | 1 |
|  |  |  |  | Dominant | 245/138 | 234/149 | 0.6742 | 0.4116 | 0.4554 | 1 |
|  |  |  |  | Recessive | 65/318 | 56/327 | 0.795 | 0.3726 | 0.4281 | 1 |
| rs7975232 | *VDR* | C | A | Genotypic | 91/184/108 | 95/181/107 | 0.1153 | 0.944 | 0.955 | 1 |
|  |  |  |  | Additive | 366/400 | 371/395 | 0.06252 | 0.8026 | 0.8026 | 1 |
|  |  |  |  | Allelic | 366/400 | 371/395 | 0.06537 | 0.7982 | 0.8379 | 1 |
|  |  |  |  | Dominant | 275/108 | 276/107 | 0.006466 | 0.9359 | 1 | 1 |
|  |  |  |  | Recessive | 91/292 | 95/288 | 0.1136 | 0.7361 | 0.8005 | 1 |
| rs1544410 | *VDR* | A | G | Genotypic | 70/174/139 | 66/182/135 | 0.3558 | 0.837 | 0.8448 | 1 |
|  |  |  |  | Additive | 314/452 | 314/452 | 0 | 1 | 1 | 1 |
|  |  |  |  | Allelic | 314/452 | 314/452 | 0 | 1 | 1 | 1 |
|  |  |  |  | Dominant | 244/139 | 248/135 | 0.09091 | 0.763 | 0.8211 | 1 |
|  |  |  |  | Recessive | 70/313 | 66/317 | 0.143 | 0.7053 | 0.7768 | 1 |
| rs2228570 | *VDR* | T | C | Genotypic | 65/156/162 | 46/162/175 | 3.867 | 0.1446 | 0.1456 | 1 |
|  |  |  |  | Additive | 286/480 | 254/512 | 2.685 | 0.1013 | 0.1013 | 1 |
|  |  |  |  | Allelic | 286/480 | 254/512 | 2.929 | 0.08703 | 0.09731 | 1 |
|  |  |  |  | Dominant | 221/162 | 208/175 | 0.8954 | 0.344 | 0.3824 | 1 |
|  |  |  |  | Recessive | 65/318 | 46/337 | 3.803 | 0.05115 | 0.06431 | 0.6138 |
| rs11568820 | *VDR* | A | G | Genotypic | 34/150/209 | 21/156/206 | 0.3393 | 0.8439 | 0.8511 | 1 |
|  |  |  |  | Additive | 198/568 | 198/568 | 0 | 1 | 1 | 1 |
|  |  |  |  | Allelic | 198/568 | 198/568 | 0 | 1 | 1 | 1 |
|  |  |  |  | Dominant | 174/209 | 177/206 | 0.04733 | 0.8278 | 0.8847 | 1 |
|  |  |  |  | Recessive | 24/359 | 21/362 | 0.2125 | 0.6449 | 0.759 | 1 |
| rs4646536 | *CYP27B1* | G | A | Genotypic | 27/134/222 | 64/122/197 | 17.1 | 0.000194 | 0.000164 | 0.00213 |
|  |  |  |  | Additive | 188/578 | 250/516 | 10.4 | 0.001259 | 0.001259 | 0.01637 |
|  |  |  |  | Allelic | 188/578 | 250/516 | 12.29 | 0.000455 | 0.000554 | 0.00592 |
|  |  |  |  | Dominant | 161/222 | 186/197 | 3.293 | 0.06958 | 0.08143 | 0.853 |
|  |  |  |  | Recessive | 27/356 | 64/319 | 17.07 | 0.036 | 0.0478 | 0.000432 |
| rs3782130 | *CYP27B1* | C | G | Genotypic | 4/143/236 | 48/121/214 | NA | NA | 1.17E-07 | 0.000002 |
|  |  |  |  | Additive | 151/615 | 217/549 | 14.76 | 0.000122 | 0.000122 | 0.001591 |
|  |  |  |  | Allelic | 151/615 | 217/549 | 15.58 | 0.0791 | 0.0983 | 1 |
|  |  |  |  | Dominant | 147/236 | 169/214 | NA | NA | 0.1232 | 1 |
|  |  |  |  | Recessive | 4/379 | 48/335 | NA | NA | 3.46E-08 | 4.5E-07 |
| rs10877012 | *CYP27B1* | T | G | Genotypic | 25/139/219 | 28/124/231 | 1.345 | 0.5103 | 0.5102 | 1 |
|  |  |  |  | Additive | 189/577 | 180/586 | 0.2725 | 0.6017 | 0.6017 | 1 |
|  |  |  |  | Allelic | 189/577 | 180/586 | 0.2892 | 0.5908 | 0.6327 | 1 |
|  |  |  |  | Dominant | 164/219 | 152/231 | 0.7757 | 0.3785 | 0.4195 | 1 |
|  |  |  |  | Recessive | 25/358 | 28/355 | 0.1824 | 0.6693 | 0.7761 | 1 |
| rs703842 | *CYP27B1* | C | T | Genotypic | 25/140/218 | 27/123/233 | 1.675 | 0.4329 | 0.4403 | 1 |
|  |  |  |  | Additive | 190/576 | 177/589 | 0.5726 | 0.4492 | 0.4492 | 1 |
|  |  |  |  | Allelic | 190/576 | 177/589 | 0.6056 | 0.4365 | 0.4726 | 1 |
|  |  |  |  | Dominant | 165/218 | 150/233 | 1.213 | 0.2707 | 0.3039 | 1 |
|  |  |  |  | Recessive | 25/385 | 27/356 | 0.08253 | 0.7739 | 0.8859 | 1 |
| rs4809957 | *CYP24A1* | G | A | Genotypic | 15/134/234 | 25/123/235 | 2.973 | 0.2262 | 0.2259 | 1 |
|  |  |  |  | Additive | 164/602 | 173/593 | 0.3014 | 0.583 | 0.583 | 1 |
|  |  |  |  | Allelic | 164/602 | 173/593 | 0.3081 | 0.5788 | 0.6218 | 1 |
|  |  |  |  | Dominant | 149/234 | 148/235 | 0.005499 | 0.9409 | 1 | 1 |
|  |  |  |  | Recessive | 15/368 | 25/258 | 2.638 | 0.1044 | 0.143 | 1 |
| rs6068816 | *CYP24A1* | T | C | Genotypic | 13/67/303 | 12/87/284 | 3.252 | 0.1967 | 0.2138 | 1 |
|  |  |  |  | Additive | 93/673 | 111/655 | 1.623 | 0.2027 | 0.2027 | 1 |
|  |  |  |  | Allelic | 93/673 | 111/655 | 1.832 | 0.1759 | 0.201 | 1 |
|  |  |  |  | Dominant | 80/303 | 99/284 | 2.632 | 0.1047 | 0.1242 | 1 |
|  |  |  |  | Recessive | 13/370 | 12/317 | 0.04135 | 0.8389 | 1 | 1 |
| For Additive Model and Allelic Model, the counts shown in "Cases" and "Controls" are allele counts, not genotype counts, and for Genotypic Model, Dominant Model, and Recessive Model, the counts shown in "Cases" and "Controls" are genotype counts. Chr: Chromosome; FET: Fisher’s exact test; NA: Not Applicable; a: *p*-value for Bonferroni correction. Shade means the result is significant or it shows a tendency. | | | | | | | | | | |
