# Supplementary material for: Molecular study of vitamin D metabolism-related single nucleotide polymorphisms in cardiovascular risk: a case-control study
Source: J Physiol Biochem. 2025 Apr 16;81(2):347–57. doi: 10.1007/s13105-025-01080-z (PMC12279573; doi:10.1007/s13105-025-01080-z)
Supplement: Supplementary file 1 — Supplementary Material 1 [file 13105_2025_1080_MOESM1_ESM.zip › Table S7.docx]

**Table S7. Influence of *CYP27B1* rs4646536 and *CYP27B1* rs3782130 gene polymorphisms on the risk of developing cardiac arrhythmias.**

| **Models** | **Genotype** | **Cases [n (%)]** | **Controls**  **[n (%)]** | ***p*-value (FET)** | **OR (CI95%)** | **Adjusted**  ***p*-value^a^** |
| --- | --- | --- | --- | --- | --- | --- |
| ***CYP27B1* rs4646536** | | | | | | |
| Genotypic | AA | 86 (60.10) | 150 (52.40) | < 0.001 | 1 | 0.009 |
|  | AG | 52 (36.40) | 93 (32.50) |  | 0.97 (0.63-1.49) |  |
|  | GG | 5 (3.50) | 43 (15.00) |  | 0.21 (0.07-0.48) |  |
| Recessive | AA + AG | 138 (96.50) | 243 (85.00) | < 0.001 | 1 | 0.002 |
|  | GG | 5 (3.50) | 43 (15.00) |  | 0.20 (0.07-0.48) |  |
| Dominant | AA | 86 (60.10) | 150 (52.40) | 0.149 | 1 | 1.946 |
|  | AG + GG | 57 (39.90) | 136 (47.60) |  | 0.73 (0.48-1.09) |  |
| Allelic | A | 224 (78.32) | 393 (68.70) | 0.003 | - | 0.047 |
|  | G | 62 (21.68) | 179 (31.30) |  | - |  |
| Additive | - | - | - | 0.006 | 0.64 (0.47-0.88) | 0.080 |
| ***CYP27B1* rs3782130** | | | | | | |
| Genotypic | GG | 89 (62.20) | 163 (57.00) | 0.001 | 1 | 0.014 |
|  | GC | 52 (36.40) | 93 (32.50) |  | 1.02 (0.66-1.57) |  |
|  | CC | 2 (1.40) | 30 (10.50) |  | 0.12 (0.01-0.42) |  |
| Recessive | GG + GC | 141 (98.60) | 256 (89.50) | < 0.001 | 1 | 0.004 |
|  | CC | 2 (1.40) | 30 (10.50) |  | 0.12 (0.02-0.41) |  |
| Dominant | GG | 89 (62.20) | 163 (57.00) | 0.349 | 1 | 4.539 |
|  | GC + CC | 54 (37.80) | 123 (43.00) |  | 0.80 (0.53-1.21) |  |
| Allelic | G | 230 (80.42) | 419 (73.25) | 0.022 | - | 0.295 |
|  | C | 56 (19.58) | 153 (26.75) |  | - |  |
| Additive | - | - | - | 0.026 | 0.68 (0.48-0.95) | 0.347 |

^a^*p*-value for Bonferroni correction. Shade means the value is significant. FET: Fisher’s extract test. OR: odds ratio; CI: confidence interval.
