# Supplementary material for: Molecular study of vitamin D metabolism-related single nucleotide polymorphisms in cardiovascular risk: a case-control study
Source: J Physiol Biochem. 2025 Apr 16;81(2):347–57. doi: 10.1007/s13105-025-01080-z (PMC12279573; doi:10.1007/s13105-025-01080-z)
Supplement: Supplementary file 1 — Supplementary Material 1 [file 13105_2025_1080_MOESM1_ESM.zip › Table S8.docx]

**Table S8. Association of 13 SNPs in vitamin D metabolism genes with risk of peripheral vascular disease**

| **SNP** | **Gene** | **Minor**  **Allele** | **Major**  **Allele** | **Model** | **Cases** | **Controls** | ***p*-value**  **(FET)** | **Adjusted *p*-value^a^** |
| --- | --- | --- | --- | --- | --- | --- | --- | --- |
| rs7041 | *GC* | A | C | Genotypic | 18/39/28 | 65/191/84 | 0.199 | 1 |
|  |  |  |  | Additive | 75/95 | 321/359 | 0.449 | 1 |
|  |  |  |  | Allelic | 75/95 | 321/359 | 0.470 | 1 |
|  |  |  |  | Dominant | 57/28 | 256/84 | 0.123 | 1 |
|  |  |  |  | Recessive | 18/67 | 65/275 | 0.668 | 1 |
| rs10741657 | *CYP2R1* | A | G | Genotypic | 17/39/29 | 40/147/153 | 0.066 | 0.861 |
|  |  |  |  | Additive | 73/97 | 227/453 | 0.022 | 0.289 |
|  |  |  |  | Allelic | 73/97 | 227/453 | 0.019 | 0.255 |
|  |  |  |  | Dominant | 56/29 | 187/153 | 0.069 | 0.906 |
|  |  |  |  | Recessive | 17/68 | 40/300 | 0.046 | 0.601 |
| rs731236 | *VDR* | G | A | Genotypic | 18/41/26 | 59/156/125 | 0.506 | 1 |
|  |  |  |  | Additive | 77/93 | 274/406 | 0.246 | 1 |
|  |  |  |  | Allelic | 77/93 | 274/406 | 0.236 | 1 |
|  |  |  |  | Dominant | 59/26 | 215/125 | 0.287 | 1 |
|  |  |  |  | Recessive | 18/67 | 59/281 | 0.413 | 1 |
| rs7975232 | *VDR* | C | A | Genotypic | 3/32/50 | 20/139/181 | NA | NA |
|  |  |  |  | Additive | 38/132 | 179/501 | 0.273 | 1 |
|  |  |  |  | Allelic | 38/132 | 179/501 | 0.288 | 1 |
|  |  |  |  | Dominant | 35/50 | 159/181 | NA | NA |
|  |  |  |  | Recessive | 3/82 | 20/320 | NA | NA |
| rs1544410 | *VDR* | T | C | Genotypic | 13/41/31 | 42/144/154 | 0.331 | 1 |
|  |  |  |  | Additive | 67/103 | 228/452 | 0.157 | 1 |
|  |  |  |  | Allelic | 67/103 | 228/452 | 0.149 | 1 |
|  |  |  |  | Dominant | 54/31 | 186/154 | 0.142 | 1 |
|  |  |  |  | Recessive | 13/72 | 42/298 | 0.469 | 1 |
| rs2228570 | *VDR* | A | G | Genotypic | 18/37/30 | 91/153/96 | 0.367 | 1 |
|  |  |  |  | Additive | 73/97 | 335/345 | 0.160 | 1 |
|  |  |  |  | Allelic | 73/97 | 335/345 | 0.139 | 1 |
|  |  |  |  | Dominant | 55/30 | 244/96 | 0.202 | 1 |
|  |  |  |  | Recessive | 18/67 | 91/249 | 0.291 | 1 |
| rs11568820 | *VDR* | T | C | Genotypic | 16/43/26 | 50/154/136 | 0.254 | 1 |
|  |  |  |  | Additive | 75/95 | 254/426 | 0.109 | 1 |
|  |  |  |  | Allelic | 75/95 | 254/426 | 0.105 | 1 |
|  |  |  |  | Dominant | 59/26 | 204/136 | 0.110 | 1 |
|  |  |  |  | Recessive | 16/69 | 50/290 | 0.348 | 1 |
| rs4646536 | *CYP27B1* | G | A | Genotypic | 10/29/46 | 53/112/175 | 0.673 | 1 |
|  |  |  |  | Additive | 49/121 | 218/462 | 0.463 | 1 |
|  |  |  |  | Allelic | 49/121 | 218/462 | 0.416 | 1 |
|  |  |  |  | Dominant | 39/46 | 165/175 | 0.662 | 1 |
|  |  |  |  | Recessive | 10/75 | 53/287 | 0.374 | 1 |
| rs3782130 | *CYP27B1* | C | G | Genotypic | 9/30/46 | 25/115/200 | 0.550 | 1 |
|  |  |  |  | Additive | 48/122 | 165/515 | 0.306 | 1 |
|  |  |  |  | Allelic | 48/122 | 165/515 | 0.285 | 1 |
|  |  |  |  | Dominant | 39/46 | 140/200 | 0.431 | 1 |
|  |  |  |  | Recessive | 9/76 | 25/315 | 0.325 | 1 |
| rs10877012 | *CYP27B1* | T | G | Genotypic | 1/33/51 | 36/114/190 | NA | NA |
|  |  |  |  | Additive | 35/135 | 186/494 | 0.086 | 1 |
|  |  |  |  | Allelic | 35/135 | 186/494 | 0.072 | 0.937 |
|  |  |  |  | Dominant | 34/51 | 150/190 | NA | NA |
|  |  |  |  | Recessive | 1/84 | 36/304 | NA | NA |
| rs703842 | *CYP27B1* | G | A | Genotypic | 9/29/47 | 26/115/199 | 0.657 | 1 |
|  |  |  |  | Additive | 47/123 | 167/513 | 0.429 | 1 |
|  |  |  |  | Allelic | 47/123 | 167/513 | 0.406 | 1 |
|  |  |  |  | Dominant | 38/47 | 141/199 | 0.589 | 1 |
|  |  |  |  | Recessive | 9/76 | 26/314 | 0.377 | 1 |
| rs4809957 | *CYP24A1* | G | A | Genotypic | 4/13/68 | 10/76/254 | NA | NA |
|  |  |  |  | Additive | 21/149 | 96/584 | 0.572 | 1 |
|  |  |  |  | Allelic | 21/149 | 96/584 | 0.550 | 1 |
|  |  |  |  | Dominant | 17/68 | 86/254 | NA | NA |
|  |  |  |  | Recessive | 4/81 | 10/330 | NA | NA |
| rs6068816 | *CYP24A1* | T | C | Genotypic | 4/29/52 | 23/110/207 | NA | NA |
|  |  |  |  | Additive | 37/133 | 156/524 | 0.751 | 1 |
|  |  |  |  | Allelic | 37/133 | 156/524 | 0.743 | 1 |
|  |  |  |  | Dominant | 33/52 | 133/207 | NA | NA |
|  |  |  |  | Recessive | 4/81 | 23/317 | NA | NA |
| For Additive Model and Allelic Model, the counts shown in "Cases" and "Controls" are allele counts, not genotype counts, and for Genotypic Model, Dominant Model, and Recessive Model, the counts shown in "Cases" and "Controls" are genotype counts.  Chr: Chromosome; FET: Fisher’s exact test; NA: Not Applicable; a: *p*-value for Bonferroni correction.  Shade means the result is significant or it shows a tendency. | | | | | | | | |
