# Supplementary material for: Molecular study of vitamin D metabolism-related single nucleotide polymorphisms in cardiovascular risk: a case-control study
Source: J Physiol Biochem. 2025 Apr 16;81(2):347–57. doi: 10.1007/s13105-025-01080-z (PMC12279573; doi:10.1007/s13105-025-01080-z)
Supplement: Supplementary file 1 — Supplementary Material 1 [file 13105_2025_1080_MOESM1_ESM.zip › Table S9.docx]

**Table S9. Influence of *CYP2R1* rs10741657 gene polymorphism on the risk of peripheral vascular disease.**

| **Models** | **Genotype** | **Cases [n (%)]** | **Controls**  **[n (%)]** | ***p*-value**  **(FET)** | **OR (CI95%)** | **Adjusted**  ***p*-value^a^** |
| --- | --- | --- | --- | --- | --- | --- |
| ***CYP2R1* rs10741657** | | | | | | |
| Genotypic | GG | 29 (34.100 | 153 (45.00) | 0.066 | 0.45 (0.22-0.90) | 0.861 |
|  | GA | 39 (45.9) | 147 (43.20) |  | 0.62 (0.32-1.24) |  |
|  | AA | 17 (20.00) | 40 (11.80) |  | 1 |  |
| Recessive | GG + GA | 68 (80.00) | 300 (88.20) | 0.046 | 0.53 (0.29-1.02) | 0.601 |
|  | AA | 17 (20.0) | 40 (11.80) |  | 1 |  |
| Dominant | GG | 29 (34.10) | 153 (45.00) | 0.069 | 0.63 (0.38-1.03) | 0.906 |
|  | GA + AA | 56 (65.90) | 187 (55.00) |  | 1 |  |
| Allelic | G | 97 (57.06) | 453 (66.2) | 0.019 | - | 0.255 |
|  | A | 73 (42.94) | 227 (33.38) |  | - |  |
| Additive | - | - | - | 0.022 | 0.68 (0.48-0.95) | 0.289 |

^a^*p*-value for Bonferroni correction. Shade means the value is significant. FET: Fisher’s extract test. OR: odds ratio; CI: confidence interval.
